# *In Vitro* and *In Vivo* Antimalarial Evaluations of Myrtle Extract, a Plant Traditionally Used for Treatment of Parasitic Disorders

**DOI:** 10.1155/2013/316185

**Published:** 2013-12-23

**Authors:** Farzaneh Naghibi, Somayeh Esmaeili, Noor Rain Abdullah, Mehdi Nateghpour, Mahdieh Taghvai, Siamak Kamkar, Mahmoud Mosaddegh

**Affiliations:** ^1^Traditional Medicine and Materia Medica Research Center (TMRC), Shahid Beheshti University of Medical Sciences, P.O. Box 14155-6354, Tehran 1516745811, Iran; ^2^School of Pharmacy, Shahid Beheshti University of Medical Sciences, Tehran, Iran; ^3^School of Traditional Medicine, Shahid Beheshti University of Medical Sciences, Tehran, Iran; ^4^Herbal Medicine Research Center, Institute for Medical Research, 50588 Kuala Lumpur, Malaysia; ^5^School of Public Health, Tehran University of Medical Sciences, Tehran, Iran

## Abstract

Based on the collected ethnobotanical data from the Traditional Medicine and Materia Medica Research Center (TMRC), Iran, *Myrtus communis* L. (myrtle) was selected for the assessment of *in vitro* and *in vivo* antimalarial and cytotoxic activities. Methanolic extract of myrtle was prepared from the aerial parts and assessed for antiplasmodial activity, using the parasite lactate dehydrogenase (pLDH) assay against chloroquine-resistant (K1) and chloroquine-sensitive (3D7) strains of *Plasmodium falciparum*. The 4-day suppressive test was employed to determine the parasitemia suppression of the myrtle extract against *P. berghei* *in vivo*. The IC_50_ values of myrtle extract were 35.44 *µ*g/ml against K1 and 0.87 *µ*g/ml against 3D7. Myrtle extract showed a significant suppression of parasitaemia (84.8 ± 1.1% at 10 mg/kg/day) in mice infected with *P. berghei* after 4 days of treatment. Cytotoxic activity was carried out against mammalian cell lines using methyl thiazol tetrazolium (MTT) assay. No cytotoxic effect on mammalian cell lines up to 100 *µ*g/mL was shown. The results support the traditional use of myrtle in malaria. Phytochemical investigation and understanding the mechanism of action would be in our upcoming project.

## 1. Introduction

**Figure 1:**
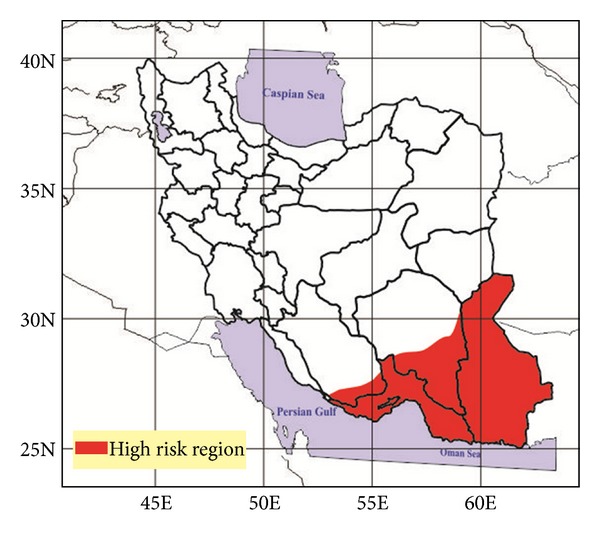
Map of malaria risk areas in Iran.

Malaria is a classic example of a disease that affects the productivity of individuals, families, and the whole society [1]. The number of deaths due to malaria was estimated to be 655000 in 2010 [2]. Malaria was highly endemic in the Caspian areas in the north and Persian Gulf littoral and plain areas in the south parts of Iran. It had been widely prevalent for a long time in the country [3]. At present the southern area of Iran (Figure 1), accommodating numerous emigrants including Afghanis and Pakistanis, is considered a high risk region in the area. According to the Ministry of Health of Iran the total number of malaria cases in Iran had been estimated to be 2900 cases in 2010 [4].

A dramatic recrudescence of malaria is ongoing due to the increasing resistance of mosquito vectors to insecticides and resistance of parasites, mainly *Plasmodium falciparum*, to available modern drugs [5]. Since malaria chemotherapy is complicated by drug-resistant strains of *Plasmodium*, new antimalarial agents are needed [6]. Traditional treatments may well prove to be the source of new antimalarial agents in view of the success with the two important chemotherapeutic agents, quinine and artemisinin, both of which are derived from plants [1]. Iran has an honorable past in traditional medicine. One of the most significant ancient heritages is sophisticated experience of people who have tried over millennia to find useful plants for health improvement, with each generation adding its own experience to this tradition [7].

This study is based on the ethnomedicinal data of the indigenous people of Kohgiluyeh-va-Boyer Ahmad province of Iran (unpublished data), where aqueous extract of myrtle is known as antiparasitic agent. Myrtle has been previously reported for various ailments like gastric ulcer, diarrhea, dysentery, vomiting, rheumatism, hemorrhage, deep sinuses, leucorrhoea, and cosmetic purposes [8]. In this study the *in vitro* and *in vivo* antiplasmodial activities and *in vitro *cytotoxic effect of the plant were assessed. The methanolic extract was selected for this experiment avoiding possible elimination of active compounds.

## 2. Materials and Methods

### 2.1. Collection and Extraction

*Myrtus communis *L. was collected in its natural habitats. Botanical identification was performed by voucher specimen (TMRC 1169) deposited at the herbarium of the Traditional Medicine and Materia Medica Research Center (TMRC). The aerial parts of the plant were allowed to dry in shadow until desiccated. Plant sample was crushed into powder using a hammer mill and stored at room temperature in appropriate container.

10 g of powdered dried aerial parts was macerated in methanol with constant shaking at the room temperature for 24 hours. The filtrate was evaporated to dryness and used for further assessments.

### 2.2. *In Vitro* Antiplasmodial Activity

*In vitro* screens for compound activity require the ability to culture *P. falciparum *in human erythrocytes [9]. The chloroquine-resistant (K1) and chloroquine-sensitive (3D7) strains of *P. falciparum *were continuous subcultured *in vitro* from cryopreservation and maintained in human red blood cells, diluted to 7% hematocrit with RPMI1640 medium. All cultures were placed in the candle jar at 37°C under 3% O_2_, 6% CO_2_, and 91% N_2_ atmosphere.

*In vitro* antiplasmodial activity, following the lactate dehydrogenase (LDH) method was assessed [10]. *Plasmodium *species depend on LDH for the metabolism of carbohydrates. Parasite LDH(pLDH) is used for the conversion of lactate into pyruvate, which is the last step in glycolysis; however, only pLDH can use coenzyme 3-acetyl-pyridine adenine dinucleotide (APAD). At the presence of APAD, the detection of LDH is specific for the parasite enzyme. LDH determination is carried out in the presence of nitro blue tetrazolium (NBT) which is reduced to formazan that is detected at 630 nm.

Starting concentration of chloroquine diphosphate and artemisinin which served as positive controls was 20 mg/mL. Myrtle extract was dissolved in DMSO to produce stock solution of 20 mg/mL. The stock solutions were subsequently diluted with deionized water at twenty different concentrations. Twofold serial dilutions were made in 96-well microtitre plates in duplicate and infected erythrocytes were added to give a 2% hematocrit and 1% parasitaemia. For the infected control, parasitized red blood cells were devoid of myrtle extract, whereas only nonparasitised red blood cells were prepared for noninfected control. Test samples were incubated at 37°C for 24 hours and subsequently cooled at −20°C to lyse the red blood cells. The plates were next allowed to reach room temperature. The spectrophotometric assessment of LDH activity was facilitated by adding 20 *μ*L nitroblue tetrazolium (NBT) and phenazine ethosulfate mixture to the 100 *μ*L MALSTAT reagent. Absorbance was measured with an ELISA plate reader at 630 nm. The percentage inhibition at each concentration was determined and the mean of the IC_50_ values of parasite viability was calculated using probit analysis [11].

### 2.3. *In Vitro *Cytotoxicity Activity

Preferably, the extract for antiplasmodial investigation should have no cytotoxicity. So the cytotoxicity of myrtle was measured by the colorimetric methyl thiazol tetrazolium (MTT) assay and scored as a percentage of absorbance reduction at 570 nm of treated cultures versus untreated control cultures.

MCF7 (breast adenocarcinoma), HepG2 (hepatocellular carcinoma), WEHI (fibrosarcoma), and MDBK (normal kidney cells) were seeded into 96-well microplates at 10^4^ cells per well and allowed to grow for 24 hours. The initial concentration of extract was 100 *μ*g/mL in DMSO, which was serially diluted in complete culture medium with two fold dilutions. Different concentrations of the extract were added to each well. Plates were incubated at 37°C for 72 hours under 5% CO_2_ atmosphere. Then the 50 *μ*L of MTT-PBS solution in culture medium was added to each well. The plates were further incubated for 4 hours at the same condition. The medium was then removed and replaced with 200 *μ*L of DMSO to solubilize the MTT formazan product. The solutions were shaked for 20 min and the absorbance at 570 nm was measured. The IC_50_ values were calculated from the drug concentration-response curves. Tamoxifen was used as a positive control with concentrations from 50 to 1.56 *μ*g/mL.

### 2.4. *In Vivo* Antimalarial Assay

The suppressive activity of the methanol extract of myrtle was assessed using the 4-day suppressive test against *Plasmodium berghei *infection in mice [12]. All the procedure was accepted by Shahid Beheshti University Ethics committee and in accordance with the principles for laboratory animal use and care in the European community guidelines. Since the advantage of intraperitoneal injection is to be very easy to perform in comparison with other routes of administration, this route was selected for the study. Adult male albino mice, weight 30 ± 3 g, were inoculated with *P. berghei, *and each mouse received 1 × 10^7^ infected erythrocytes by intraperitoneal injection on the first day of the experiment. The mice were randomly divided into experimental test and control consisting of 5 mice per cage. Two control groups were used in this experiment: one was treated with chloroquine at dose of 20 mg/kg as a positive control while the other group was kept untreated given normal saline as placebo. The mice of test group were treated during consecutive day with 10 mg/kg of the sample by intraperitoneal injection for 4 days. On days 5, 8, and 15 of the test, thin blood smears were made from the tail blood of the mice. The blood films were fixed with methanol and stained with Giemsa and then assessed by microscope. Percentage of parasitaemia was counted based on infected erythrocytes calculated per 1000 erythrocytes.

## 3. Results

Myrtle extract was prepared from the aerial part of *Myrtus communis *and was first tested at 20 concentrations on the *Plasmodium falciparum *K1 and 3D7 strains using pLDH assay. The cytotoxic activity was performed against three cancer cell lines (MCF7, HepG2, and WEHI) and normal cell line (MDBK). The 4-day suppressive test against *P. berghei *was carried out. The results of the *in vitro *and *in vivo *antiplasmodial and cytotoxic activities of the *Myrtus communis *are shown in Table 1. Myrtle extract showed better antiplasmodial activity against sensitive 3D7 strain with no cytotoxic activity up to 100 *μ*g/mL against the selected cell lines.

**Figure 2:**
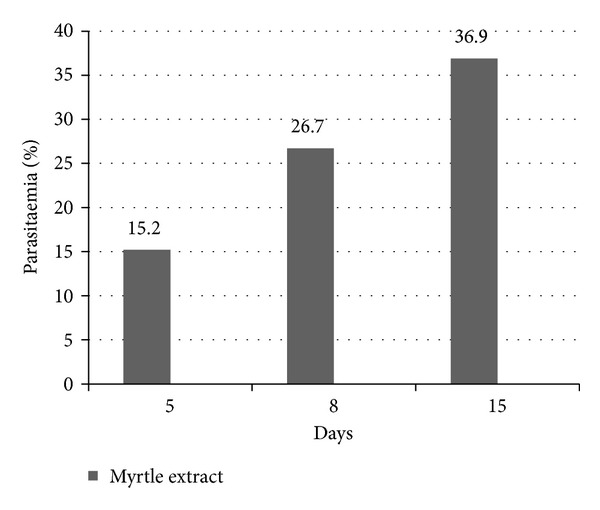
Percentage of parasitaemia in the test group (myrtle extract) on days 5, 8, and 15 in comparison with placebo.

Percentage of parasitaemia on days 5, 8, and 15 of the experiment in the test group in comparison with placebo group is shown in Figure 2. The average survival days of the test group were 18 ± 0.57 days. All the mice of placebo group died within two weeks of experiment.

## 4. Discussion

Our previous studies [13, 14] and this report lead us to carry out more surveys in Iranian folk medicine and traditional medicine which may help to detect new effective plants. The aim of this survey was the assessment of *in vitro* and *in vivo* antimalarial activities and *in vitro* cytotoxic effect of a plant traditionally used for treatment of parasitic infections.

Based on ethnobotanical data of some provinces of Iran that was carried out at TMRC [13, 14] and a study which revealed potential antiplasmodial activity of essential oil of *Myrtus communis* L. [15], myrtle was selected for this survey.

*Myrtus communis* L. is an aromatic and medicinal species from the Myrtaceae family. Myrtle is used in folk medicine of Iran for treatment of some diseases such as parasitic disorders and herpes [14, 16, 17].

Ideally, effective extracts at the blood stage of the malaria parasite should have strong *in vitro *and* in vivo* antimalarial activities and should be devoid of cytotoxicity at concentration up to 100 *μ*g/mL [18]. Peters test is an appropriate method used to assessment *in vivo *antiplasmodial activity of plant extracts. Myrtle has strong *in vitro* antiplasmodial activity (IC_50_ = 35.44 and 0.87 *μ*g/mL) with no cytotoxicity up to 100 *μ*g/mL (Table 1). After four days of treatment the suppressive percentage of parasitaemia with 84.8% was obtained for the methanolic extract of myrtle. This result is interesting when it was compared with the results from other plant extracts reported in the following literatures works.

Antimalarial activity of different extract doses of *Cocos nucifera* was investigated *in vivo* against *Plasmodium berghei* (NK65) infections in mice. Chemosuppression effects of 44.71%, 56.86%, 79.61%, and 83.73% were, respectively, shown for the corresponding dose of extract (50, 100, 200, and 400 mg/kg) [19]. In another study ethanolic leaf extract of *Verbena hastata *was evaluated using chloroquine-sensitive *P. berghei berghei *infection in mice at various doses (200, 400, and 800 mg/kg) causing 64%, 70%, and 71% chemosuppression, respectively [20].

Chloroform extracts of *Artemisia macrivera* Linn. and *A. maritime* Linn. showed antimalarial activity in mice against *P. berghei* NK65 at dose of 100 mg/kg with average percentage parasitaemia 0.30 ± 0.04 and 0.40 ± 0.05, respectively, on day 5 of the test [21].

Methanolic extracts from 15 medicinal plants in Kenya were screened for their *in vivo *antimalarial activity in mice against a chloroquine-tolerant *P. berghei *NK65 at a dose of 500 mg/kg. The best percentage of suppression on day 4 was 59.3% for *Toddalia asiatica *[22]. Other workers have studied the *in vivo *antimalarial activity of the methanol extract of *Annona senegalensis *against *P. berghei *at the doses of 100, 200, 400, 600, and 800 mg/kg. Chemosuppression of parasitaemia was 57.1%, 59.3%, 76.3%, 89.8%, and 91.1%, respectively [23]. The extracts of *Cassia occidentalis*, *Morinda morindoides,* and *Phyllanthus niruri *were evaluated for their antimalarial activity *in vivo *against *P. berghei *ANKA in mice at dose of 200 mg/kg. The most active extract that was from *Morinda morindoides* reduced parasitaemia by 74% [24]. Extracts obtained from the leaf and stem of *Quassia amara *and* Q. undulate *were screened against *P. berghei berghei *in mice at doses of 100 and 200 mg/kg. The parasite density (%) was between 0.6 and 8.5 on day 5 [25].

For comparison among some plant extracts that were screened for their *in vivo *antiplasmodial activity using Peters method, myrtle was shown to have dramatic effects at a low dose (10 mg/kg) in day four. Despite the decreasing suppression of parasitaemia on days 8 and 15, still chemosuppression of myrtle extract with 63.1% was observed.

Myrtle extract has compounds such as monoterpenoids, flavonoides, triterpenoids, and phloroglucinol type compounds [26]. Phytochemical investigation and isolation of various compounds from *Myrtus communis* has led to the identification of some compounds like *β*-sitosterol, myricetin, myricitrin, myrtillin, chrysanthemin, oenin, delphidin-3-arabinoside, cyanidin-3-arabinoside, petunidin-3-glucoside, petunidin-3-arabinoside, peonidin-3-glucoside, malvidin-3-arabinoside, 3-methoxymyricetin-7-O-*α*-L-rhamnopyranoside, and myrtucommulones. Among these compounds myricitrin isolated from the aerial part of *Euphorbia hirta* exhibited antiplasmodial activity with IC_50_ value of 5.4 *μ*g/mL against *P. falciparum* [27]. This compound isolated from the leaves of *Licania octandra* possessed antiplasmodial activity (IC_50_ = 17.37 *μ*g/mL) [28]. Myricetin is another compound isolated from edible plants and showed antiplasmodial activity against *P. falciparum* 3D7 and 7G8. The IC_50_ values were 40 *μ*g/mL and 76 *μ*g/mL, respectively [29]. Furthermore, myricetin was found to have antimalarial activity when tested against *P. falciparum* K1 and NF54 with IC_50_ values of 12.9 *μ*g/mL and 57.3 *μ*g/mL [30]. Among the other myrtle compounds, *β*-sitosterol isolated from the leaves of *Teclea trichocarpa* displayed antiplasmodial activity with IC_50_ value of 8.20 *μ*g/mL against *P. falciparum* K1 [31].

Although several classes of natural products are responsible for the antiplasmodial activity of many plant species used in traditional medicine for the treatment of malaria, the most important and diverse biopotency has been observed in alkaloids, quassinoids, and sesquiterpene lactone. Nonalkaloidal natural compounds from plants with antiplasmodial and antimalarial properties, belonging to the classes of terpenes, limonoids, flavonoides, chromone, xanthone, anthraquinone, and related compounds, were recently reviewed [32]. According to this, flavonoides and steroides from the myrtle might have antiplasmodial activity, and bioassay guided fractionation resulting in isolated active components of *Myrtus communis* is necessary.

## 5. Conclusion

To our knowledge, myrtle extract has not been previously studied for its antiplasmodial activity. Our evaluation of the plant against two strains of *Plasmodium falciparum in vitro* and *P. berghei in vivo* proved antimalarial activities with no cytotoxicity up to 100 *μ*g/mL. The results suggest that the Iranian ethnic medicinal application of myrtle has a pharmacological basis. Phytochemical investigation and also understanding the mechanism of action would be the next step of this study.

## Figures and Tables

**Table 1:** *In vitro *and *in vivo *antiplasmodial activity and cytotoxic effect on mammalian cell lines.

Sample	*In vitro *antiplasmodial activity of *Plasmodium falciparum* IC_50_ (*μ*g/mL)		Cytotoxicity IC_50_ (*μ*g/mL)				*In vivo* antiplasmodial activity of *Plasmodium berghei *%* *inhibition
	K1	3D7	MCF7	HepG2	WEHI	MDBK	
*Myrtus communis* L.	35.44	0.87	>100	>100	>100	>100	84.8 ± 1.1 (day 5) 63.1 ± 4.1 (day 15)
Chloroquine	0.02	0.01	23.90	34.60	12.29	>100	100
Artemisinin	0.001	0.004	>100	>100	>100	>100	N.D
Tamoxifen	N.D.	N.D	3.60	4.38	19.1	6.35	N.D

N.D.: not done.
